# Editorial

**DOI:** 10.1038/sj.bjc.6601940

**Published:** 2004-06-08

**Authors:** R A Weiss

**Affiliations:** Editor-in-Chief

Alert readers, authors and reviewers of papers in BJC will have noticed new names among our Subject Editors. Jim Cassidy (University of Glasgow) has joined us as Clinical Editor, Richard Houlston (Institute of Cancer Research, Sutton) now edits papers in Genetics and Genomics, and Bryan Leyland-Jones (McGill University, Montreal) has just begun to edit Experimental Therapeutics. Moreover, Chris Boshoff is now properly acknowledged as our Reviews Editor, a task he has been doing unassumingly, with vision, keen judgement, and good taste, since we initiated our series of minireviews 3 years ago.

These changes mean that it is time to relieve three old friends of their duties: Robert Hawkins (University of Manchester) has been our Clinical Editor for rather more than 8 years, John Double (University of Bradford) has looked after Experimental Therapeutics for almost as long, and Carlo Croce (Thomas Jefferson University, Philadelphia) began to help develop the then new Genetics and Genomics Section 3 years ago. We are immensely grateful for their hard work and for their very fine evaluation and editorship of papers. They will be much missed as colleagues and for the intellectual contribution that they have made to the success of BJC as a wide-ranging, cutting-edge journal in the expanding and increasingly busy arena of cancer research. I am particularly indebted to Robert Hawkins for facilitating considerable growth in the number and quality of clinical papers submitted to BJC, and for the increasingly lively engagement of the clinical community via letters, editorials, and mini-reviews on topics ranging from original research findings to policy matters. We heartily welcome this development and hope to see it continue. John Double built upon the heritage of my predecessor, the late Ged Adams. They made BJC the natural home of some of the very best papers, taking promising research findings through experimental systems and pharmacology to establish that they had potential for improved treatment for patients. The BJC welcomes and encourages papers from those who work with ‘translation’ as their goal. Moreover, John has ever been mindful that while the use of animals is crucial to advances in some areas of cancer research, BJC publishes only those papers reporting research that meets stringent standards of ethics and animal welfare.

Our outgoing Subject Editors have set high standards that I know our new colleagues are keen to uphold and enhance. Supported by sophisticated on-line systems, we have been able to improve the speed at which papers are reviewed and published. BJC intends to continue in these directions and to enhance the publication service that it offers to the cancer research community. At the heart of our activities, we have a large number of dedicated regular reviewers, to whom we owe immeasurable thanks; only a very few of them can be listed on the Editorial Board. Peer review remains the pivotal means of selecting and honing the best research, and we are deeply indebted and grateful to all our colleagues who spend valuable hours on this process.

